# A Study on Tool Breakage Detection Technology Based on Current Sensing and Non-Contact Signal Analysis

**DOI:** 10.3390/s25133880

**Published:** 2025-06-22

**Authors:** Chia-Hung Lai, Sih-Hao Huang, Ting-En Wu, Chia-Chun Lai

**Affiliations:** 1 Department of Intelligent Automation Engineering, National Chin-Yi University of Technology, Taichung 411030, Taiwan; chlai@ncut.edu.tw (C.-H.L.); 3b261070@gm.student.ncut.edu.tw (S.-H.H.); 2 Department of Aeronautics and Astronautics, National Cheng Kung University, Tainan 701, Taiwan; 3 Office of Intelligent Engineering Virtual-Real Learning (OIEVR), National Taichung University of Science and Technology, Taichung 404, Taiwan

**Keywords:** tool condition monitoring, current sensing, non-contact monitoring, predictive maintenance, deep learning

## Abstract

**Highlights:**

**What are the main findings?**

- A non-contact tool breakage monitoring method based on spindle current sensing signals is proposed.
- The accuracy and computational efficiency of ANN, DNN, and CNN models are verified and compared.

**What is the implication of the main finding?**

- Applicable to machining environments where vibration sensors cannot be installed.
- Enables the real-time monitoring of tool status without hardware modification, facilitating predictive maintenance.

**Abstract:**

Tool breakage in CNC machining often leads to reduced productivity and increased maintenance costs. This study proposes a non-contact tool breakage detection method using spindle current signals captured by an SCT013 current sensor. The sensor easily attaches to the motor line without any hardware modification and provides real-time current signals for frequency domain analysis. Fast Fourier Transform (FFT) is employed to extract spectral features, particularly focusing on high-frequency energy spikes at the moment of breakage. A total of 20 experiments were conducted, and consistent spectral anomalies were observed. Additionally, deep learning models including ANN, DNN, and CNN were compared for automated detection performance. The results indicate that the proposed system can reliably detect tool breakage by identifying frequency domain anomalies that emerge within 1–3 s after the actual event, based on processed current signals. While the inference time of deep learning models ranges from 15 to 58 s, the detection mechanism captures the breakage characteristics early in the signal, enabling timely tool condition evaluation.

## 1. Introduction

In Computer Numerical Control (CNC) machining systems, tool breakage is a common and high-risk unexpected event. It can interrupt production processes, lead to workpiece scrapping, and even cause equipment damage or safety hazards. Therefore, the real-time and accurate monitoring of tool usage status has become a critical issue in intelligent manufacturing. To meet this demand, Tool Condition Monitoring (TCM) technologies have continuously drawn attention from both academia and industry.

Mohanraj et al. [1] reviewed various sensor technologies applied to tool wear and breakage detection. Among them, dynamometers offer high-precision force measurements and can reflect cutting force changes in real time, but they are costly and complex to install. Accelerometers are easy to install and offer wide frequency response but are susceptible to structural vibration and environmental interference. In contrast, current sensors are widely adopted in industrial practice due to their ease of installation and low cost. However, current signals may be affected by background noise such as motor startups and relay switching, leading to misjudgments.

Xu et al. [2] used vibration sensors to capture signals during the cutting process and applied Support Vector Machines (SVMs) to classify tool breakage, proving its classification capability. However, such vibration sensors must be installed on the spindle or work platform, and their installation space and procedures are often restricted, limiting their practical application. Zhang et al. [3] proposed a vision or optical-based detection method, which provides high recognition accuracy. Still, in environments with cutting fluid or chips, the tool must be moved to a fixed sensing position, interrupting the machining process and prolonging operation time. Gerdes et al. [4] and Santoro et al. [5] demonstrated the potential of active thermography for impact damage detection in composite materials, highlighting its advantages in speed and cost-effectiveness. However, the present study focuses on micro-tools with a diameter of 0.3 mm, where thermal imaging techniques may face limitations in resolution and responsiveness for detecting sudden breakage events at such small scales.

Given these limitations, recent studies have favored non-contact current sensing as an alternative method for tool condition inference. Peng et al. [6] observed stable periodic features in current signals under specific spindle operating states using voltage and current sensors. Miura et al. [7] estimated actual cutting current through corrected voltage and current signals, effectively correlating with cutting loads. We adopt the SCT013 sensor as an external current measurement device. Its quick clip-on installation, non-destructive nature, and excellent anti-interference performance enable the real-time monitoring of spindle current changes without modifying mechanical structures or control circuits.

To improve signal discrimination accuracy, Lee et al. [8] implemented a low-pass filter (LPF) at the circuit input to suppress background noise and enhance system stability. This design serves as a preprocessing foundation for subsequent frequency domain feature extraction. Regarding feature extraction, Reñones et al. [9] proposed constructing statistical feature vectors based on noise, vibration, temperature, and power consumption. Sun et al. [10] used an SVM for tool breakage classification and achieved effective results. Wu et al. [11] applied a Symmetrized Dot Pattern (SDP) combined with a Convolutional Neural Network (CNN) to classify gear defects, achieving an accuracy above 91%.

Inspired by these studies, we adopt high-frequency energy spikes in the frequency domain as recognition features and compare three deep learning models—an Artificial Neural Network (ANN), a Deep Neural Network (DNN), and a Convolutional Neural Network (CNN)—to evaluate their classification performance and computational cost. Lai et al. [12] also integrated time domain, frequency domain, and time frequency analyses (e.g., DWT and STFT) into the deep learning frameworks to enhance the recognition capability of various mechanical faults, which serves as a structural reference for our study.

Furthermore, Reeber et al. [13] noted that internal current signals from CNC controllers have limited frequency response. Using external sensors can capture higher-frequency components and improve real-time anomaly detection. Rüppel et al. [14] and Miura et al. [15] explored load variation and output power estimation based on Multi-Sample Rate (MSR) structures and feed drive current. Peng et al. [16] applied a data-driven regression model to successfully map current variations to cutting force characteristics.

In summary, this study proposes a current sensing-based, non-contact tool breakage detection system that allows real-time monitoring without modifying existing CNC hardware. It effectively identifies breakage anomalies and demonstrates strong potential for applications in smart manufacturing and predictive maintenance.

## 2. Theoretical Framework

### 2.1. Current Signal Processing

Before capturing current signals, preprocessing is applied to remove noise interference and ensure the accuracy of subsequent feature extraction. To eliminate high-frequency noise that may affect signal stability, a low-pass filter is used to retain the primary energy band of the signal. The low-pass filter is calculated as shown in Equation (1). Additionally, since the analog input voltage on the Arduino must remain within a specified range, a DC offset is designed to prevent overflow, as shown in Equation (2).

(1) $$f_{c}=\frac{1}{2\pi RC}$$

where $f_{C}=Cut-off\;frequency,R=Resistance,C=Capacitance$

(2) $$C_{out}=C_{in}\frac{R1}{R1+R2}$$

where $C_{out}=Output\;voltage,C_{in}=Input\;voltage,R1=Resistance1,R2=Resistance2$.

Since spindle current is an AC signal, it must be converted into an equivalent DC energy representation. For example, Lin et al. [17] used Root Mean Square (RMS), crest factor, and kurtosis to extract features. In this study, we calculate the RMS current as shown in Equation (3) and use the peak-to-peak value to assess spike characteristics, as in Equation (4).

(3) $$I_{RMS}=\sqrt{\frac{1}{N}\sum_{n-1}^{N}{\left(X(n)\right)}^{2}}$$

where $I_{RMS}=Root\;Mean\;Square$, N=Total number of samples, $X\left(n\right)=Current\;value\;at\;the\;n\;sampling\;point.$

(4) $$I_{pp}=I_{max}-I_{min}$$

where $I_{pp}=Peak\;to\;peak\;value$, $I_{max}=Maximum\;current\;value$, $I_{min}=Minimum\;current\;value$.

### 2.2. Fast Fourier Transform (FFT)

To further analyze the frequency domain characteristics of current signals during the machining process, Fast Fourier Transform (FFT) is employed to convert time domain signals into their corresponding frequency domain representations. FFT is an efficient numerical implementation of the Fourier Transform (FT), as shown in Equation (5), and enables rapid frequency analysis of continuous or discrete signals, thereby enhancing signal processing efficiency. Compared to other time frequency analysis techniques such as Short-Time Fourier Transform (STFT) and Discrete Wavelet Transform (DWT), FFT provides a more concise and computationally efficient approach, particularly suitable for capturing the high-frequency energy spikes associated with sudden tool breakage events under stable cutting conditions. In scenarios where tracking temporal frequency evolution is unnecessary, FFT stands out as a simple yet effective method for detecting abrupt spectral anomalies.

To reduce spectral leakage during the FFT process, the Hanning window function is applied for signal weighting. The number of sampling points per operation is set to 512 (*n* = 512), resulting in a frequency resolution of approximately 2 Hz, as shown in Equation (6). With this configuration, the system can clearly identify sudden changes in high-frequency energy. By comparing the frequency domain features of normal machining and breakage events, abnormal spikes or energy concentration anomalies can be preliminarily identified—serving as key indicators for evaluating tool conditions.

(5) $$X\left(k\right)=\sum_{n=0}^{N-1}X\left(n\right)e^{-j\frac{2\pi }{N}kn},\;0\leq k\leq N-1$$

where X(k) = the frequency domain value at index k, X(n) = the time domain sample at index n, *j* = an imaginary unit, and *N* = signal length.

(6) $$∆f=\frac{f_{s}}{N}$$

where $∆f=Frequency\;resolution,\;f_{s}=Sampling\;frequency$, N=Number of FFT sampling points.

### 2.3. Deep Learning Models (ANN, DNN, and CNN)

To predict tool breakage moments, we draw from Huang et al. [18], who proposed a method combining Discrete Wavelet Transform (DWT) and Deep Neural Networks (DNNs) for defect detection in surface-modified gears. They successfully identified vibration features under different failure states. Therefore, we further compare three types of neural networks for tool breakage detection performance: ANN, DNN, and CNN. The goal is to evaluate recognition accuracy and analyze differences in detection time, parameters, and training cost among these models.

#### 2.3.1. Artificial Neural Network (ANN)

**Figure 1:**
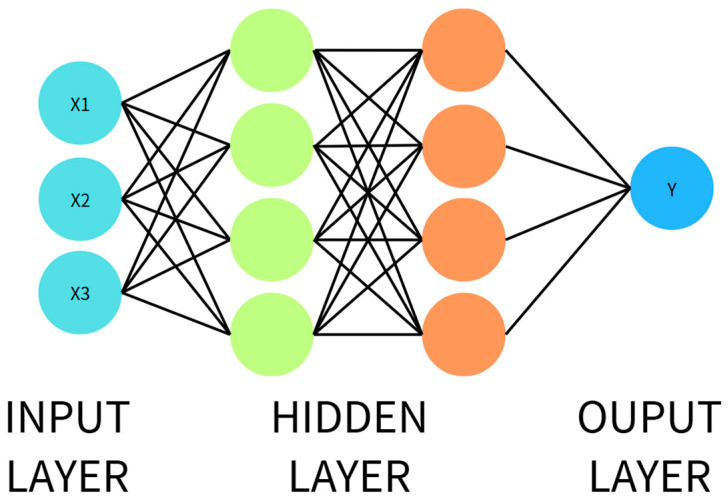
Structure of an ANN.

An ANN simulates the connection mechanism of biological neurons and is composed of input, hidden, and output layers. Each neuron transmits input information to the next layer through weights and applies a non-linear transformation via an activation function. An ANN is suitable for structured data and can learn the mapping between input features and output classes. However, its performance is limited for complex data, requiring deeper architectures (e.g., DNN) for enhancement. The ANN schematic is shown in Figure 1, and its operation is described in Equation (7).

The ANN model was trained with a learning rate of 0.001. The training process was conducted over 20 epochs with a batch size of 32. The network architecture included one hidden layer containing 128 neurons, followed by a dropout layer with a rate of 0.3 to reduce the risk of overfitting. The hidden layer used the ReLU activation function, while the output layer used the sigmoid activation function to perform binary classification.

(7) $$Z=\sum_{i=1}^{n}w_{i}x_{i}+b\;\;\;\;\;a=f(z)$$

where $x_{i}=i^{th}\;input\;value\;w_{i}=Weight\;corresponding\;b=Bias\;value\;in\;the\;neuron\;a=output.$

#### 2.3.2. Deep Neural Network (DNN)

**Figure 2:**
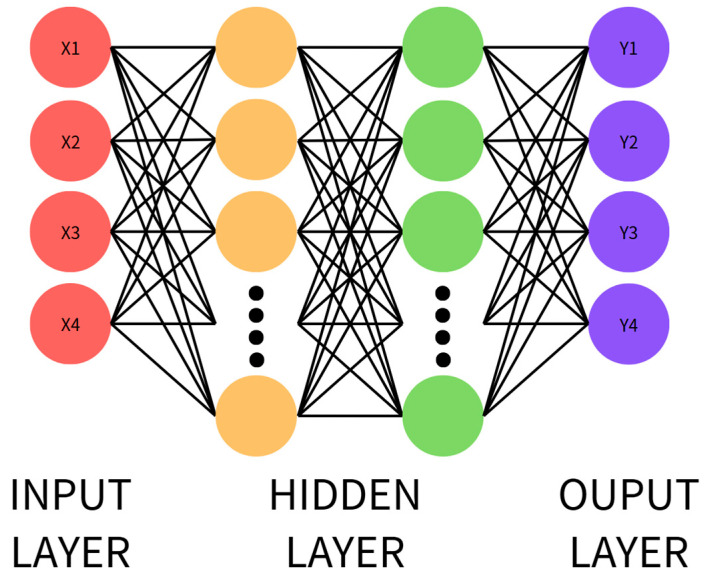
Structure of a DNN.

A DNN is an extension of an ANN, characterized by multiple hidden layers, which provide strong feature extraction and high-level semantic understanding. With deep stacking and non-linear transformations, a DNN can learn complex feature relationships and achieve high accuracy with sufficient data. It is particularly suitable for FFT-preprocessed feature vectors, such as current energy and amplitude. The DNN schematic is shown in Figure 2, with the computational process described in Equation (8).

The DNN model was trained with a learning rate of 0.001. The training process was conducted over 20 epochs with a batch size of 32. The network architecture consisted of three hidden layers, containing 256, 128, and 64 neurons, respectively. After each hidden layer, a dropout layer with rates of 0.4, 0.3, and 0.3 was applied to prevent overfitting. The hidden layers used the ReLU activation function, while the output layer employed the sigmoid activation function to perform binary classification.

(8) $$Z^{\left[l\right]}=W^{\left[l\right]}a^{\left[l\right]}+b^{\left[l\right]}$$

where $\left[l\right]=Index\;of\;the\;l\;layer\;W^{\left[l\right]}=Weight\;matrix\;of\;the\;l\;layer,\;b^{\left[l\right]}=Bias\;vector\;a^{\left[l\right]}=Output\;of\;the\;l\;layer.$

#### 2.3.3. Convolutional Neural Network (CNN)

A CNN is a deep learning model commonly used for image and time series data. It can automatically identify local features in the data, such as regions with significant changes or specific patterns. In this study, we transform the FFT-processed current signals into spectrogram-like images, which are then analyzed by the CNN to learn and detect abnormal patterns related to tool breakage.

**Figure 3:**
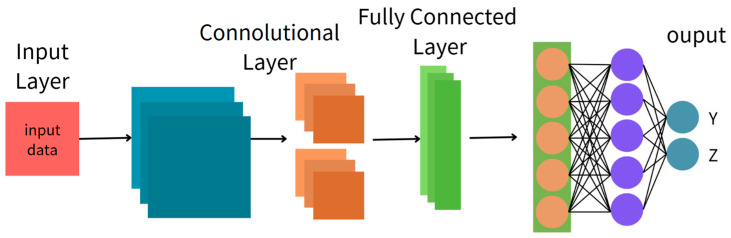
Structure of a CNN.

The CNN model used in this study consists of three convolutional layers. Each layer uses a small 3 × 3 filter to scan the data (with a stride of 1). After each convolutional block, a 2 × 2 max pooling layer is applied to reduce the data size while preserving important features. The output of each layer is passed through a ReLU activation function for non-linear transformation, and a dropout layer is added during training to prevent overfitting. The overall structure is shown in Figure 3, and the processing flow corresponds to Equation (9).

(9) $$s\left(t\right)=\left(x*w\right)\left(t\right)=\sum_{\tau =0}^{k-1}x\left(t+\tau \right)w(\tau )$$

where x=Input image or feature vector w=Corresponding weight k=Convolution kernel.

## 3. Materials and Methods

### 3.1. Experimental Framework and Sensor Configuration

**Figure 4:**
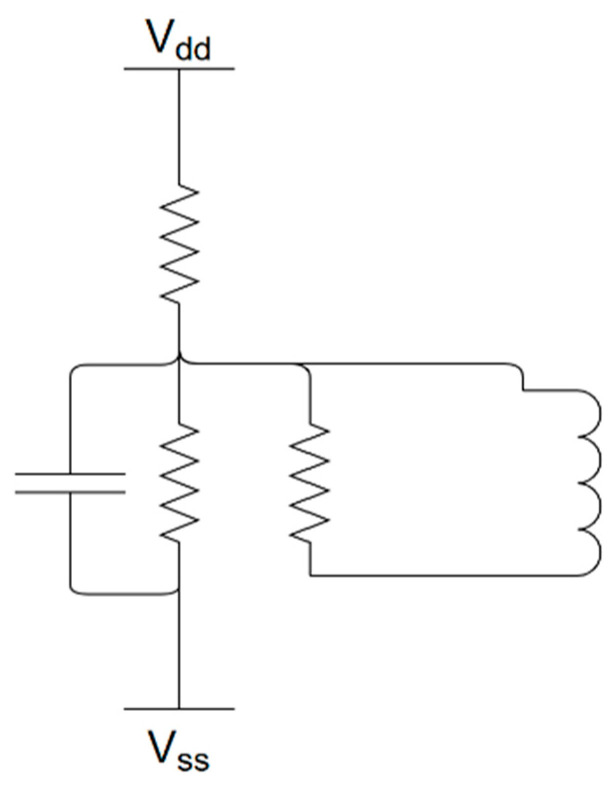
SCT013 Wiring Diagram.

**Figure 5:**
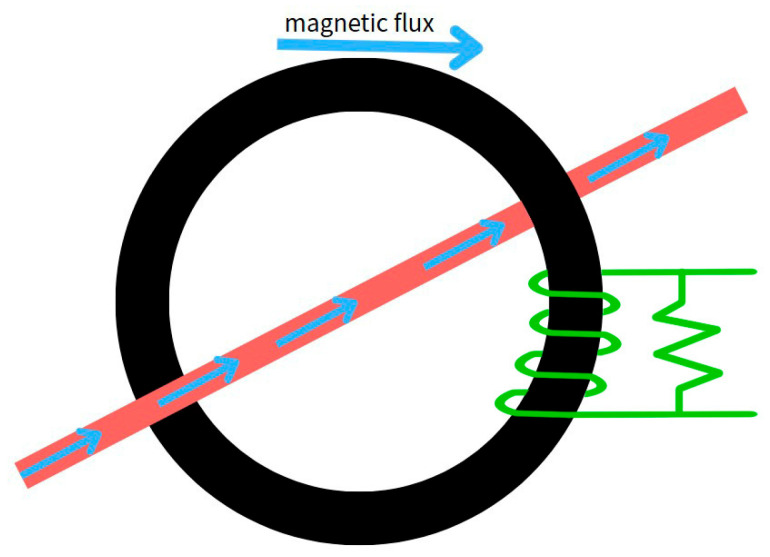
Electromagnetic Induction Schematic.

To conduct tool breakage monitoring experiments, we designed a non-contact monitoring system based on spindle current. The current signal is primarily captured using an SCT013 current sensor, as shown in the wiring diagram in Figure 4. This sensor is a type of split-core current transformer (CT) that operates based on the principle of electromagnetic induction, illustrated in Figure 5. It can be directly clamped onto the motor power cable without interrupting machine operation or requiring any hardware or wiring modifications, thereby enabling non-intrusive measurement. The sensor outputs a voltage signal proportional to the current, which is then divided and filtered before being sent to a data acquisition device for storage and analysis.

To ensure system safety and accuracy, the output current from the CT is limited. In this study, an SCT013 sensor (100 A–1 V) is used in the experiment due to its frequency response range of approximately 50 Hz to 1 kHz and a response time of less than 100 milliseconds, making it suitable for detecting transient high-frequency features generated by sudden tool breakage. In addition, a DC bias adjustment is applied to keep the output voltage within ± 5 V to prevent damage to the receiving device. To further improve signal clarity, a low-pass filter and DC offset correction are applied as part of the preprocessing procedure to suppress background noise caused by motor startups and relay switching. Repeated experiments showed that high-frequency spikes in the FFT consistently appeared at the same breakage moments, indicating strong anti-interference capability of the system.

The experimental platform consists of a PD-5 high-speed engraving and milling machine equipped with a 0.03 mm carbon steel drill bit. Machining tests are performed at a spindle speed of 20,000 RPM and a cutting speed of 1.884 m/min. The SCT013 sensor, with a maximum current capacity of 30 A, is used to monitor the current signal, which is sampled at a rate of 1 kHz.

### 3.2. Experimental Procedure

First, the current signals captured during the tool machining process undergo preprocessing, including DC offset correction and low-pass filtering, to remove background noise and voltage interference, ensuring that the signals input into the controller are stable and identifiable.

After preprocessing, the signals are retained in the time domain and also transformed into the frequency domain using Fast Fourier Transform (FFT) to capture spectral variations at the moment of tool breakage. These two types of features are then used to construct a complete training dataset.

**Figure 6:**
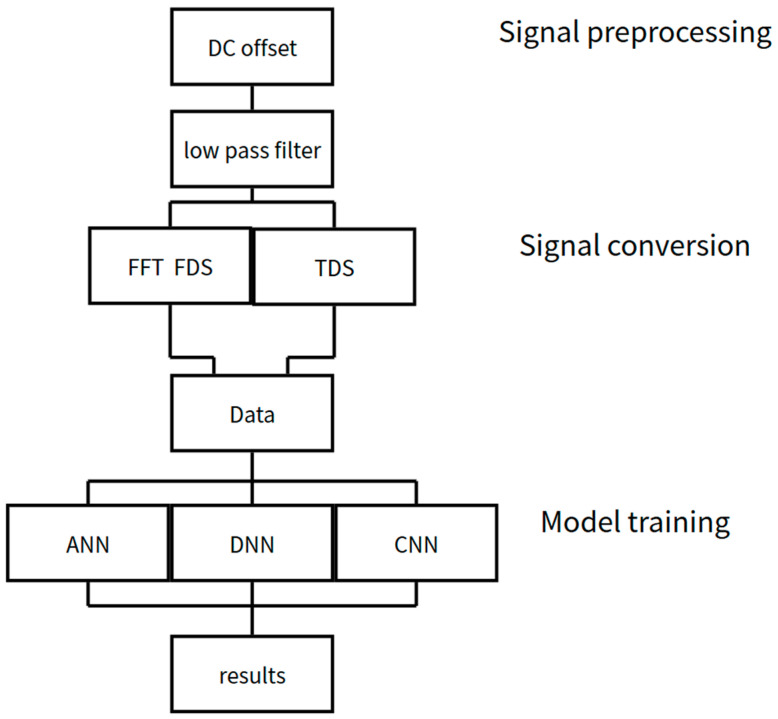
Experimental Flowchart.

The dataset is then input into deep learning models. In this study, we compare three architectures—ANN, DNN, and CNN—to evaluate their differences in performance and train them to classify whether tool breakage or anomalies have occurred. Finally, the trained models output predictions regarding the tool condition. The complete workflow is illustrated in Figure 6.

### 3.3. Model Training and Dataset Preparation

A total of 20 normal machining samples were collected, each lasting approximately 40 s, representing stable cutting conditions. In addition, 40 ERROR samples were generated by simulating tool breakage events under controlled conditions. To prepare the dataset for training, all 60 samples were labeled and split into training, validation, and testing sets in a 7:2:1 ratio. Stratified sampling was applied to maintain class distribution across all subsets, ensuring that each set contained both normal and ERROR data. This method reduces the risk of class imbalance during training, which is critical for reliable learning in binary classification tasks. Ensuring that each subset reflects the actual class proportions improves model robustness, prevents overfitting to the majority class, and enhances generalization performance when exposed to unseen scenarios in real-world deployment.

## 4. Results and Discussion

### 4.1. Time and Frequency Domain Analysis of Current Signal

This study conducted a total of 20 experiments. Each experiment was divided into two phases:

Phase 1 involved normal machining operation lasting approximately 10 s to establish a stable baseline signal.

Phase 2 involved simulating a tool breakage event, either by suddenly removing the cutting load or lightly striking the tool, to trigger a breakage signal.

**Figure 7:**
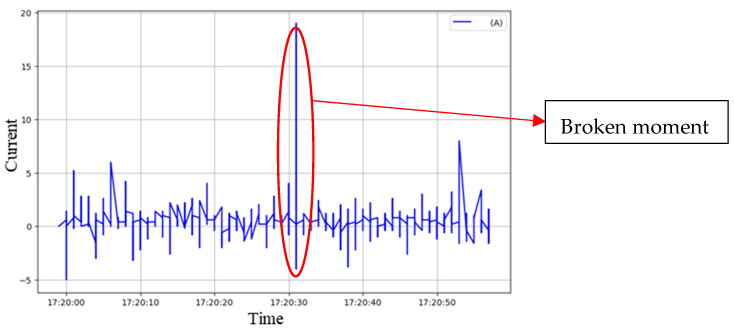
Current Signal–Time Domain (Tool Breakage Highlighted).

During each simulated breakage event, the operator simultaneously pressed a timestamp button, marking the corresponding signal as the Ground Truth for training and evaluation. All breakage simulations were performed by the same operator under identical cutting conditions, using standardized triggering actions (e.g., consistent tapping angle and force) to ensure experimental consistency and reproducibility. Figure 7 shows the time domain variation in the current signal from one of the experiments. A significant disturbance can be observed at approximately 17:20:31, corresponding to the tool breakage event recorded by the operator. Some smaller current fluctuations are also present, caused by external factors such as relay switching. However, these background fluctuations did not produce prominent high-frequency energy spikes in the subsequent frequency domain analysis and were therefore not misclassified as breakage events. The system relies primarily on the presence of sharp high-frequency spikes as the key distinguishing feature, allowing it to effectively differentiate tool breakage signals from normal background variations and thereby enhancing classification robustness.

**Figure 8:**
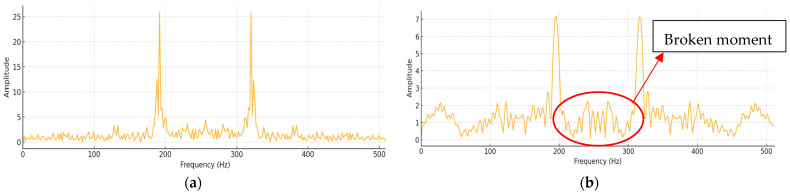
The frequency domain results obtained. (**a**) Spectrum under normal processing conditions. (**b**) Frequency domain diagram of tool breakage.

Figure 8 presents the frequency domain spectra (FFT results) obtained from this experiment. Subfigure (a) shows the spectrum under normal machining conditions, while subfigure (b) depicts the spectrum at the moment of tool breakage. A comparison between the two reveals significant spectral changes, particularly the emergence of sharp high-frequency energy spikes during breakage. These spike features were consistently observed in more than 18 out of 20 experiments, confirming their high reliability as indicators of tool breakage. However, it is essential to eliminate background disturbances—such as those caused by motor startups and relay switching—prior to frequency domain analysis to ensure accurate feature extraction.

**Figure 9:**
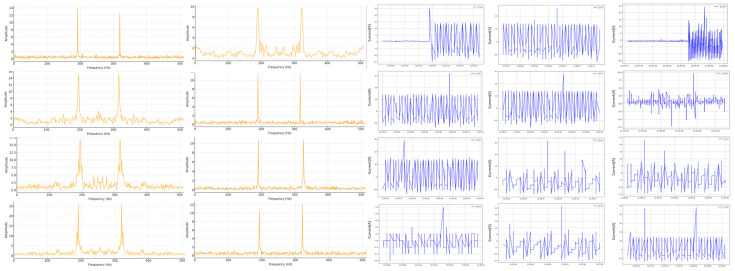
Frequency domain spectra from other experimental conditions.

Figure 9 additionally illustrates frequency domain spectra from other experimental conditions, providing supplementary evidence of high-frequency behavior during tool breakage under varied scenarios, further reinforcing the consistency of the observed spectral features.

### 4.2. System Detection Accuracy and Latency Analysis

Three deep learning models—ANN, DNN, and CNN—were applied to classify tool breakage based on current signals. Their accuracy and inference time were compared. The model-predicted breakage times were matched against the manually recorded timestamps.

From frequency domain analysis, CNN achieved the highest accuracy (98.1%), but its inference time was nearly twice as long as DNN. Considering both performance and computational efficiency, DNN provided the best balance. The comparison is shown in Table 1.

In contrast, ANN performed more steadily in time domain predictions due to its lightweight structure and faster inference, though its accuracy was slightly lower. Still, the differences in prediction probability among the three models were minimal. The summarized results for time domain predictions are shown in Table 2.

Additionally, three iterations of training were conducted using 5-fold cross-validation. A line chart of performance trends and a histogram of the best-performing model can be drawn to illustrate the findings.

Table 1 summarizes the performance of the three deep learning models (ANN, DNN, CNN) in classifying tool breakage based on current signals.

Section (a) presents overall performance metrics, including Accuracy, Precision, Recall, F1 score, and Inference Time, providing a comprehensive comparison of model performance and computational efficiency.

Section (b) displays the confusion matrices of ANN, DNN, and CNN, offering detailed insights into each model’s classification behavior, particularly the distribution of True Positive and False Negative cases, as a reference for classification reliability.

The comparison shows that CNN achieved the highest accuracy but incurred a relatively longer inference time. DNN provided the best balance between accuracy and computational efficiency, representing the most favorable model overall. ANN demonstrated stable performance in time domain predictions and the fastest inference time, though with slightly lower accuracy. The confusion matrices further indicate that all models maintained low misclassification rates across various testing scenarios, demonstrating robust classification performance.

## 5. Conclusions

Based on the above analysis results, the following conclusions are obtained:

This study proposes a non-contact tool breakage detection technology that utilizes an SCT013 current sensor to capture CNC spindle current signals, combined with FFT-based frequency domain energy analysis as the basis for identifying tool breakage events. The system requires no modification to existing CNC machine structures, offers easy installation and low cost, and is particularly suitable for space-constrained environments or applications sensitive to equipment modification. Experimental results demonstrate that tool breakage can be detected within 1–3 s, and three deep learning models (ANN, DNN, CNN) were evaluated for classification performance, with the DNN model achieving the best balance between accuracy and computational efficiency. The proposed approach can enhance micro-tool condition monitoring and predictive maintenance in smart manufacturing environments, helping to reduce production disruption risks and improve process stability, thereby showing strong potential for industrial applications.

(1) FFT features need to be customized for different machine types and cutting parameters. Future work will focus on standardizing energy threshold definitions and conducting sensitivity analyses to improve feature robustness.
(2) The current dataset was collected from a single machine under fixed cutting conditions. Future studies will expand data collection to multiple machines, tool types, and materials to enhance model generalization and industrial applicability.
(3) The models have only been validated offline and have not been compared with traditional methods. Future work will incorporate threshold-based approaches and statistical metrics such as MAE, MSE, and SNR to provide a more comprehensive performance evaluation.
(4) Although the CNN model achieves the highest accuracy, its inference time is too long for real-time applications. Future research will explore pruning, quantization, and edge computing techniques to accelerate inference and enable real-time deployment.
(5) Further reducing latency beyond the current 1–3 s detection window to better protect machinery, especially in time-critical machining scenarios, is desirable.
(6) A fixed 1 s signal windowing strategy was used, and no data augmentation was applied. Future work will investigate augmentation techniques to improve model robustness.

## Figures and Tables

**Table 1:** Performance Metrics of Deep Learning Models on Frequency Domain Features.

Model (K-Fold-5)	Accuracy (%)	Precision	Recall	F1-Score	Inference Time (S)
ANN	0.925	0.91	0.93	0.92	16
DNN	0.946	0.93	0.95	0.94	26
CNN	0.953	0.94	0.96	0.95	58
(a) Comparison of frequency domain performance indicators					
Confusion Matrix	True Positive	False Positive	True Negative	False Negative	
ANN	235	15	230	20	
DNN	240	10	234	16	
CNN	234	7	238	12	
(b) Frequency Domain Confusion Matrix					

**Table 2:** Time domain performance indicators of deep learning models.

Model (K-Fold-5)	Accuracy (%)	Precision	Recall	F1-Score	Inference Time (S)
ANN	97.6	0.96	0.98	0.97	15
DNN	97.8	0.97	0.99	0.98	23
CNN	98.1	0.98	0.99	0.99	44
(a) Comparison of frequency domain performance indicators					
Confusion Matrix	True Positive	False Positive	True Negative	False Negative	
ANN	248	2	242	8	
DNN	249	1	243	7	
CNN	250	0	245	5	
(b) Time Domain Confusion Matrix					

## Data Availability

Data are contained within the article.

## Abbreviations

CNC	Computer Numerical Control
TCM	Tool Condition Monitoring
LPF	Low-Pass Filter
SVM	Support Vector Machines
SDP	Symmetrized Dot Pattern
ANN	Artificial Neural Network
DNN	Deep Neural Network
CNN	Convolutional Neural Network
FFT	Fast Fourier Transform
FFT Amp	FFT Amplitude
SCT	Split Core Transformer
LPF	Low-Pass Filter
DC	Direct Current
Hz	Hertz
A/D	Analog-to-Digital
AI	Artificial Intelligence
RMS	Root Mean Square
DC Offset	Direct Current Offset Removal
